# Supplementary material for: Hot spot prediction in protein-protein interactions by an ensemble system
Source: BMC Syst Biol. 2018 Dec 31;12(Suppl 9):132. doi: 10.1186/s12918-018-0665-8 (PMC6311905; doi:10.1186/s12918-018-0665-8)
Supplement: Supplementary file 2 — All relevant MATLAB code. A simple Matlab implement of our predictor. (ZIP 19,742 kb) [file 12918_2018_665_MOESM2_ESM.zip › Additional file 2/Read ASA/ASA website/dbMPIKT/4YH7A.html]

 NetSurfP - Protein Surface Accessibility and Secondary Structure Predictions

  

|  |  |  |
| --- | --- | --- |
|  |  | NetSurfP - Protein Surface Accessibility and Secondary Structure Predictions  Technical University of Denmark |

  


---

```
# For publication of results, please cite:
# A generic method for assignment of reliability scores applied to solvent accessibility predictions.
# Bent Petersen, Thomas Nordahl Petersen, Pernille Andersen, Morten Nielsen and Claus Lundegaard
# BMC Structural Biology 2009, 9:51 doi:10.1186/1472-6807-9-51
#
# Column 1: Class assignment - B for buried or E for Exposed - Threshold: 25% exposure, but not based on RSA
# Column 2: Amino acid
# Column 3: Sequence name
# Column 4: Amino acid number
# Column 5: Relative Surface Accessibility - RSA
# Column 6: Absolute Surface Accessibility
# Column 7: Z-fit score for RSA prediction
# Column 8: Probability for Alpha-Helix
# Column 9: Probability for Beta-strand
# Column 10: Probability for Coil
E E  4YH7A                  1    0.873 152.443   1.072   0.003   0.003   0.994
E T  4YH7A                  2    0.521  72.249   0.284   0.005   0.045   0.951
E P  4YH7A                  3    0.343  48.643   0.578   0.018   0.047   0.935
B P  4YH7A                  4    0.109  15.439  -0.382   0.021   0.279   0.699
E R  4YH7A                  5    0.471 107.767   1.073   0.002   0.816   0.182
B F  4YH7A                  6    0.144  28.841   0.088   0.001   0.900   0.099
B T  4YH7A                  7    0.256  35.493   0.267   0.002   0.816   0.182
E R  4YH7A                  8    0.456 104.378   0.332   0.004   0.616   0.381
E T  4YH7A                  9    0.416  57.727   0.779   0.004   0.197   0.799
B P  4YH7A                 10    0.218  30.906  -0.591   0.005   0.045   0.951
E V  4YH7A                 11    0.585  89.853   0.269   0.004   0.085   0.910
E D  4YH7A                 12    0.578  83.347  -0.573   0.005   0.336   0.660
B Q  4YH7A                 13    0.175  31.219   1.317   0.002   0.816   0.182
E T  4YH7A                 14    0.464  64.357   1.125   0.001   0.900   0.099
B G  4YH7A                 15    0.051   4.022   0.290   0.001   0.900   0.099
E V  4YH7A                 16    0.451  69.365   1.287   0.002   0.816   0.182
E S  4YH7A                 17    0.406  47.618   0.447   0.005   0.262   0.733
E G  4YH7A                 18    0.709  55.798  -0.759   0.005   0.045   0.951
E G  4YH7A                 19    0.446  35.108   0.772   0.004   0.085   0.910
E V  4YH7A                 20    0.519  79.693   0.995   0.004   0.616   0.381
B A  4YH7A                 21    0.037   4.099  -0.265   0.001   0.959   0.040
E S  4YH7A                 22    0.354  41.489   1.803   0.001   0.959   0.040
B F  4YH7A                 23    0.041   8.289   0.717   0.001   0.959   0.040
E I  4YH7A                 24    0.366  67.636   1.671   0.001   0.959   0.040
B C  4YH7A                 25    0.025   3.510   0.762   0.001   0.959   0.040
E Q  4YH7A                 26    0.443  79.102   1.553   0.001   0.959   0.040
B A  4YH7A                 27    0.061   6.689  -0.577   0.001   0.900   0.099
E T  4YH7A                 28    0.517  71.639   0.426   0.003   0.718   0.279
E G  4YH7A                 29    0.337  26.538  -1.905   0.005   0.262   0.733
E D  4YH7A                 30    0.537  77.411   0.350   0.004   0.138   0.858
E P  4YH7A                 31    0.268  37.958  -0.856   0.004   0.138   0.858
E R  4YH7A                 32    0.535 122.423   0.825   0.004   0.138   0.858
B P  4YH7A                 33    0.072  10.203  -0.979   0.004   0.514   0.481
E K  4YH7A                 34    0.414  85.098   1.299   0.001   0.900   0.099
B I  4YH7A                 35    0.033   6.068  -0.007   0.001   0.959   0.040
E V  4YH7A                 36    0.278  42.682   1.726   0.006   0.962   0.032
B W  4YH7A                 37    0.038   9.259  -0.214   0.006   0.962   0.032
E N  4YH7A                 38    0.277  40.597   1.203   0.001   0.959   0.040
B K  4YH7A                 39    0.224  46.097   0.961   0.003   0.718   0.279
E K  4YH7A                 40    0.583 119.923  -0.271   0.005   0.045   0.951
E G  4YH7A                 41    0.772  60.717  -1.541   0.005   0.015   0.979
E K  4YH7A                 42    0.485  99.785   0.508   0.003   0.718   0.279
E K  4YH7A                 43    0.504 103.735   1.071   0.002   0.816   0.182
B V  4YH7A                 44    0.044   6.747  -0.265   0.021   0.451   0.528
E S  4YH7A                 45    0.466  54.592   0.800   0.004   0.197   0.799
E N  4YH7A                 46    0.613  89.787  -1.196   0.004   0.085   0.910
E Q  4YH7A                 47    0.607 108.374  -1.989   0.005   0.045   0.951
E R  4YH7A                 48    0.547 125.286  -1.541   0.004   0.085   0.910
E F  4YH7A                 49    0.363  72.754  -0.997   0.004   0.514   0.481
E E  4YH7A                 50    0.307  53.650   0.036   0.002   0.816   0.182
B V  4YH7A                 51    0.243  37.334  -0.532   0.001   0.959   0.040
B I  4YH7A                 52    0.201  37.130   0.226   0.001   0.959   0.040
E E  4YH7A                 53    0.332  58.053  -0.376   0.001   0.959   0.040
B F  4YH7A                 54    0.201  40.381  -0.193   0.001   0.900   0.099
E D  4YH7A                 55    0.527  75.897  -0.920   0.004   0.616   0.381
E D  4YH7A                 56    0.536  77.252  -1.530   0.004   0.085   0.910
E G  4YH7A                 57    0.558  43.938  -2.394   0.005   0.015   0.979
E S  4YH7A                 58    0.495  58.037  -0.924   0.004   0.197   0.799
B G  4YH7A                 59    0.242  19.053  -0.189   0.004   0.616   0.381
B S  4YH7A                 60    0.063   7.419  -0.091   0.002   0.816   0.182
B V  4YH7A                 61    0.111  17.061   0.110   0.001   0.959   0.040
B L  4YH7A                 62    0.029   5.310   0.073   0.001   0.959   0.040
B R  4YH7A                 63    0.195  44.586   1.146   0.001   0.959   0.040
B I  4YH7A                 64    0.032   5.846  -0.167   0.001   0.900   0.099
E Q  4YH7A                 65    0.356  63.653   1.045   0.002   0.816   0.182
E P  4YH7A                 66    0.504  71.589  -0.548   0.004   0.514   0.481
B L  4YH7A                 67    0.241  44.090  -0.864   0.005   0.262   0.733
E R  4YH7A                 68    0.557 127.622  -0.241   0.005   0.262   0.733
E T  4YH7A                 69    0.448  62.193  -0.055   0.004   0.197   0.799
E P  4YH7A                 70    0.515  73.036  -0.541   0.004   0.138   0.858
E R  4YH7A                 71    0.599 137.102  -0.647   0.004   0.138   0.858
B D  4YH7A                 72    0.185  26.615  -0.910   0.005   0.262   0.733
E E  4YH7A                 73    0.423  73.898  -0.007   0.004   0.420   0.576
B A  4YH7A                 74    0.090   9.929  -0.743   0.002   0.816   0.182
E I  4YH7A                 75    0.345  63.751   1.927   0.001   0.959   0.040
B Y  4YH7A                 76    0.034   7.180   0.594   0.001   0.959   0.040
B E  4YH7A                 77    0.213  37.176   2.085   0.001   0.959   0.040
B C  4YH7A                 78    0.014   1.938   1.692   0.001   0.959   0.040
E V  4YH7A                 79    0.233  35.874   2.032   0.001   0.959   0.040
B A  4YH7A                 80    0.020   2.259   0.757   0.001   0.959   0.040
E S  4YH7A                 81    0.310  36.285   0.897   0.001   0.959   0.040
B N  4YH7A                 82    0.098  14.391  -0.423   0.003   0.718   0.279
E N  4YH7A                 83    0.578  84.648  -0.009   0.004   0.138   0.858
E V  4YH7A                 84    0.430  66.091  -0.863   0.004   0.138   0.858
E G  4YH7A                 85    0.470  36.997  -1.603   0.004   0.420   0.576
E E  4YH7A                 86    0.520  90.774  -0.774   0.004   0.616   0.381
B I  4YH7A                 87    0.298  55.038  -0.768   0.003   0.718   0.279
E S  4YH7A                 88    0.476  55.822  -0.335   0.002   0.816   0.182
B V  4YH7A                 89    0.201  30.878   0.373   0.002   0.816   0.182
E S  4YH7A                 90    0.368  43.106   0.295   0.002   0.816   0.182
B T  4YH7A                 91    0.060   8.322  -0.056   0.001   0.900   0.099
E R  4YH7A                 92    0.284  65.105   1.256   0.001   0.959   0.040
B L  4YH7A                 93    0.031   5.676  -0.009   0.001   0.959   0.040
B T  4YH7A                 94    0.216  29.973   1.106   0.001   0.959   0.040
B V  4YH7A                 95    0.051   7.808   0.537   0.001   0.900   0.099
E L  4YH7A                 96    0.252  46.105   0.571   0.018   0.846   0.136
E R  4YH7A                 97    0.298  68.173  -0.071   0.023   0.655   0.322
E E  4YH7A                 98    0.377  65.827   0.229   0.020   0.205   0.775
E D  4YH7A                 99    0.485  69.889  -1.338   0.018   0.088   0.893
E Q  4YH7A                100    0.563 100.534  -1.153   0.018   0.088   0.893
E I  4YH7A                101    0.365  67.543  -1.011   0.018   0.088   0.893
E P  4YH7A                102    0.377  53.454  -1.158   0.018   0.088   0.893
E R  4YH7A                103    0.626 143.377  -2.175   0.018   0.088   0.893
E G  4YH7A                104    0.453  35.651  -1.328   0.018   0.047   0.935
B F  4YH7A                105    0.262  52.583  -0.511   0.018   0.019   0.964
B P  4YH7A                106    0.200  28.309  -1.125   0.018   0.088   0.893
E T  4YH7A                107    0.494  68.559   0.176   0.021   0.451   0.528
B I  4YH7A                108    0.095  17.538  -1.007   0.023   0.655   0.322
E D  4YH7A                109    0.417  60.075  -0.098   0.023   0.655   0.322
E M  4YH7A                110    0.403  80.640  -0.282   0.022   0.552   0.426
E G  4YH7A                111    0.488  38.374   0.207   0.004   0.197   0.799
B P  4YH7A                112    0.282  40.073  -1.376   0.004   0.085   0.910
E Q  4YH7A                113    0.572 102.088  -0.380   0.005   0.262   0.733
E L  4YH7A                114    0.522  95.487  -0.703   0.004   0.616   0.381
B K  4YH7A                115    0.239  49.080   0.657   0.002   0.816   0.182
E V  4YH7A                116    0.450  69.134   0.577   0.001   0.959   0.040
B V  4YH7A                117    0.098  15.001  -0.149   0.001   0.900   0.099
E E  4YH7A                118    0.460  80.345   0.496   0.002   0.816   0.182
E R  4YH7A                119    0.453 103.806  -0.113   0.005   0.336   0.660
E T  4YH7A                120    0.646  89.545  -0.967   0.005   0.045   0.951
E R  4YH7A                121    0.467 106.920   0.508   0.004   0.085   0.910
E T  4YH7A                122    0.483  67.006   0.492   0.004   0.514   0.481
B A  4YH7A                123    0.041   4.573  -0.003   0.001   0.900   0.099
E T  4YH7A                124    0.295  40.889   1.459   0.001   0.959   0.040
B M  4YH7A                125    0.037   7.344   0.402   0.001   0.959   0.040
E L  4YH7A                126    0.356  65.239   1.422   0.001   0.959   0.040
B C  4YH7A                127    0.030   4.156   0.393   0.001   0.900   0.099
E A  4YH7A                128    0.469  51.684   1.328   0.001   0.900   0.099
B A  4YH7A                129    0.082   9.047  -0.472   0.002   0.816   0.182
E S  4YH7A                130    0.551  64.624  -0.093   0.004   0.420   0.576
E G  4YH7A                131    0.485  38.170  -2.001   0.004   0.085   0.910
E N  4YH7A                132    0.486  71.106  -0.402   0.005   0.045   0.951
E P  4YH7A                133    0.355  50.360  -0.988   0.005   0.045   0.951
E D  4YH7A                134    0.531  76.503   0.214   0.004   0.085   0.910
B P  4YH7A                135    0.076  10.728  -1.500   0.004   0.420   0.576
E E  4YH7A                136    0.386  67.522   1.002   0.001   0.900   0.099
B I  4YH7A                137    0.057  10.527   0.264   0.001   0.959   0.040
E T  4YH7A                138    0.268  37.172   1.203   0.001   0.959   0.040
B W  4YH7A                139    0.048  11.568   0.166   0.001   0.900   0.099
E F  4YH7A                140    0.269  53.928   0.647   0.001   0.900   0.099
E K  4YH7A                141    0.271  55.786   0.461   0.004   0.616   0.381
E D  4YH7A                142    0.612  88.204  -1.544   0.018   0.019   0.964
E F  4YH7A                143    0.713 143.099  -2.367   0.018   0.019   0.964
E L  4YH7A                144    0.475  86.973  -0.538   0.004   0.616   0.381
E P  4YH7A                145    0.498  70.624   0.010   0.004   0.616   0.381
B V  4YH7A                146    0.108  16.615  -0.731   0.022   0.552   0.426
E D  4YH7A                147    0.470  67.684  -0.180   0.021   0.451   0.528
E T  4YH7A                148    0.431  59.724  -1.490   0.020   0.205   0.775
E S  4YH7A                149    0.434  50.853  -1.911   0.019   0.141   0.840
E N  4YH7A                150    0.463  67.812  -1.886   0.020   0.205   0.775
E N  4YH7A                151    0.484  70.916  -1.214   0.022   0.359   0.619
E N  4YH7A                152    0.403  58.955  -1.422   0.021   0.451   0.528
B G  4YH7A                153    0.263  20.674  -1.012   0.021   0.451   0.528
E R  4YH7A                154    0.401  91.898  -0.771   0.023   0.655   0.322
B I  4YH7A                155    0.247  45.658  -1.485   0.023   0.655   0.322
E K  4YH7A                156    0.396  81.498  -1.106   0.022   0.552   0.426
E Q  4YH7A                157    0.439  78.477  -1.607   0.021   0.451   0.528
E L  4YH7A                158    0.371  67.875  -1.652   0.021   0.279   0.699
E R  4YH7A                159    0.541 124.003  -1.173   0.022   0.359   0.619
E S  4YH7A                160    0.468  54.908  -1.365   0.021   0.451   0.528
E E  4YH7A                161    0.410  71.575  -1.046   0.022   0.552   0.426
E S  4YH7A                162    0.385  45.169  -1.244   0.023   0.655   0.322
B I  4YH7A                163    0.250  46.176  -0.904   0.003   0.718   0.279
E G  4YH7A                164    0.443  34.833  -1.646   0.003   0.718   0.279
E G  4YH7A                165    0.366  28.788  -1.115   0.002   0.816   0.182
E T  4YH7A                166    0.468  64.925  -1.428   0.004   0.616   0.381
E P  4YH7A                167    0.375  53.212  -1.577   0.005   0.262   0.733
E I  4YH7A                168    0.566 104.765  -1.577   0.004   0.197   0.799
E R  4YH7A                169    0.490 112.210  -0.516   0.005   0.336   0.660
B G  4YH7A                170    0.098   7.720  -0.478   0.004   0.616   0.381
E A  4YH7A                171    0.246  27.142   0.890   0.001   0.900   0.099
B L  4YH7A                172    0.029   5.310   0.596   0.001   0.959   0.040
E Q  4YH7A                173    0.253  45.132   1.653   0.001   0.959   0.040
B I  4YH7A                174    0.030   5.494   0.167   0.001   0.900   0.099
E E  4YH7A                175    0.435  76.047   1.398   0.002   0.816   0.182
E Q  4YH7A                176    0.494  88.175   0.287   0.004   0.616   0.381
B S  4YH7A                177    0.071   8.356  -0.408   0.005   0.262   0.733
E E  4YH7A                178    0.491  85.760   0.983   0.005   0.262   0.733
E E  4YH7A                179    0.546  95.456   0.185   0.004   0.197   0.799
E S  4YH7A                180    0.584  68.468  -0.847   0.005   0.045   0.951
E D  4YH7A                181    0.288  41.472  -0.736   0.004   0.085   0.910
E Q  4YH7A                182    0.474  84.585  -0.058   0.005   0.336   0.660
B G  4YH7A                183    0.117   9.192  -0.973   0.002   0.816   0.182
E K  4YH7A                184    0.351  72.180   1.925   0.001   0.959   0.040
B Y  4YH7A                185    0.034   7.266   0.574   0.001   0.959   0.040
B E  4YH7A                186    0.158  27.620   1.455   0.001   0.959   0.040
B C  4YH7A                187    0.012   1.713   1.829   0.001   0.959   0.040
B V  4YH7A                188    0.157  24.192   1.780   0.001   0.959   0.040
B A  4YH7A                189    0.020   2.248   0.686   0.001   0.959   0.040
B T  4YH7A                190    0.214  29.654   0.533   0.001   0.959   0.040
B N  4YH7A                191    0.100  14.684  -0.405   0.003   0.718   0.279
E S  4YH7A                192    0.514  60.288  -0.373   0.018   0.088   0.893
E A  4YH7A                193    0.430  47.419  -1.187   0.018   0.088   0.893
E G  4YH7A                194    0.387  30.425  -1.933   0.004   0.197   0.799
E T  4YH7A                195    0.538  74.648  -1.271   0.021   0.279   0.699
E R  4YH7A                196    0.464 106.325  -1.347   0.022   0.359   0.619
E Y  4YH7A                197    0.468 100.054  -1.157   0.005   0.336   0.660
E S  4YH7A                198    0.397  46.564  -1.575   0.005   0.336   0.660
E A  4YH7A                199    0.409  45.116  -0.957   0.004   0.420   0.576
E P  4YH7A                200    0.371  52.687  -0.937   0.004   0.514   0.481
B A  4YH7A                201    0.160  17.643  -1.373   0.003   0.718   0.279
E N  4YH7A                202    0.364  53.290  -0.007   0.002   0.816   0.182
B L  4YH7A                203    0.130  23.821  -0.140   0.011   0.918   0.071
E Y  4YH7A                204    0.267  57.058   0.360   0.018   0.846   0.136
B V  4YH7A                205    0.204  31.370  -0.256   0.021   0.756   0.223
E R  4YH7A                206    0.342  78.272  -0.020   0.022   0.552   0.426
E E  4YH7A                207    0.496  86.651  -1.004   0.021   0.279   0.699
E L  4YH7A                208    0.303  55.406  -0.573   0.056   0.142   0.802
E R  4YH7A                209    0.570 130.576  -0.690   0.052   0.084   0.864
E E  4YH7A                210    0.550  96.102  -1.257   0.052   0.084   0.864
E V  4YH7A                211    0.408  62.694  -1.597   0.052   0.084   0.864
E R  4YH7A                212    0.430  98.584  -1.392   0.052   0.084   0.864
E R  4YH7A                213    0.562 128.790  -1.099   0.052   0.084   0.864
E V  4YH7A                214    0.423  64.938  -0.856   0.018   0.047   0.935
E P  4YH7A                215    0.312  44.315  -0.644   0.018   0.047   0.935
B P  4YH7A                216    0.230  32.580  -1.307   0.020   0.205   0.775
E R  4YH7A                217    0.477 109.210   0.059   0.022   0.552   0.426
B F  4YH7A                218    0.126  25.208  -0.995   0.021   0.756   0.223
E S  4YH7A                219    0.350  40.961  -0.628   0.022   0.552   0.426
E I  4YH7A                220    0.446  82.418  -0.979   0.021   0.451   0.528
E P  4YH7A                221    0.438  62.110  -0.328   0.004   0.085   0.910
E P  4YH7A                222    0.316  44.784  -1.919   0.005   0.045   0.951
E T  4YH7A                223    0.495  68.601  -0.571   0.005   0.262   0.733
E N  4YH7A                224    0.473  69.218  -0.691   0.004   0.514   0.481
B H  4YH7A                225    0.196  35.652   0.776   0.002   0.816   0.182
E E  4YH7A                226    0.450  78.528   0.085   0.001   0.900   0.099
B I  4YH7A                227    0.111  20.553  -0.234   0.001   0.900   0.099
E M  4YH7A                228    0.428  85.663   0.270   0.003   0.718   0.279
E P  4YH7A                229    0.458  65.019   0.100   0.005   0.262   0.733
E G  4YH7A                230    0.622  48.959  -1.365   0.005   0.045   0.951
E G  4YH7A                231    0.425  33.432  -0.179   0.004   0.138   0.858
E S  4YH7A                232    0.509  59.690  -0.047   0.004   0.616   0.381
B V  4YH7A                233    0.075  11.574  -0.518   0.001   0.900   0.099
E N  4YH7A                234    0.411  60.200   0.805   0.001   0.900   0.099
B I  4YH7A                235    0.086  15.966   0.166   0.001   0.900   0.099
E T  4YH7A                236    0.414  57.463   0.530   0.001   0.900   0.099
B C  4YH7A                237    0.065   9.112  -0.149   0.002   0.816   0.182
E V  4YH7A                238    0.445  68.412   0.804   0.001   0.900   0.099
B A  4YH7A                239    0.078   8.585  -2.467   0.002   0.816   0.182
E V  4YH7A                240    0.489  75.175  -0.653   0.004   0.514   0.481
E G  4YH7A                241    0.477  37.548  -2.641   0.004   0.138   0.858
E S  4YH7A                242    0.500  58.577  -0.649   0.004   0.197   0.799
B P  4YH7A                243    0.226  32.098  -1.016   0.004   0.138   0.858
E M  4YH7A                244    0.402  80.460  -0.064   0.005   0.262   0.733
B P  4YH7A                245    0.127  18.064  -1.371   0.004   0.616   0.381
E Y  4YH7A                246    0.284  60.648   0.425   0.001   0.900   0.099
B V  4YH7A                247    0.046   7.039   0.240   0.001   0.959   0.040
B K  4YH7A                248    0.212  43.526   0.912   0.001   0.959   0.040
B W  4YH7A                249    0.046  11.111   0.146   0.001   0.959   0.040
B M  4YH7A                250    0.192  38.479   0.300   0.001   0.900   0.099
E L  4YH7A                251    0.279  51.012   0.053   0.003   0.718   0.279
E G  4YH7A                252    0.570  44.851  -1.720   0.004   0.085   0.910
E A  4YH7A                253    0.608  67.002  -2.566   0.018   0.019   0.964
E E  4YH7A                254    0.605 105.781  -0.890   0.005   0.336   0.660
E D  4YH7A                255    0.597  86.085  -0.220   0.004   0.420   0.576
B L  4YH7A                256    0.107  19.665  -1.042   0.005   0.262   0.733
E T  4YH7A                257    0.464  64.357  -0.214   0.004   0.138   0.858
E P  4YH7A                258    0.557  79.010  -1.510   0.004   0.197   0.799
E E  4YH7A                259    0.537  93.744  -1.345   0.005   0.262   0.733
E D  4YH7A                260    0.433  62.395  -1.251   0.004   0.514   0.481
E D  4YH7A                261    0.491  70.768  -0.733   0.003   0.718   0.279
B M  4YH7A                262    0.241  48.224  -0.449   0.003   0.718   0.279
E P  4YH7A                263    0.510  72.397  -0.656   0.004   0.616   0.381
B I  4YH7A                264    0.302  55.888  -1.750   0.005   0.262   0.733
B G  4YH7A                265    0.225  17.700  -1.362   0.004   0.197   0.799
E R  4YH7A                266    0.291  66.731  -0.796   0.004   0.616   0.381
B N  4YH7A                267    0.074  10.848  -0.750   0.002   0.816   0.182
B V  4YH7A                268    0.176  27.113   0.608   0.001   0.959   0.040
B L  4YH7A                269    0.032   5.896   0.086   0.001   0.959   0.040
E E  4YH7A                270    0.359  62.752   0.962   0.001   0.900   0.099
B L  4YH7A                271    0.266  48.760  -0.559   0.002   0.816   0.182
E N  4YH7A                272    0.327  47.917  -0.300   0.005   0.336   0.660
E D  4YH7A                273    0.525  75.710  -0.855   0.005   0.336   0.660
E V  4YH7A                274    0.325  49.922  -0.247   0.005   0.262   0.733
E R  4YH7A                275    0.513 117.500  -0.117   0.004   0.197   0.799
E Q  4YH7A                276    0.427  76.280  -0.588   0.004   0.138   0.858
E S  4YH7A                277    0.475  55.635  -1.138   0.004   0.138   0.858
B A  4YH7A                278    0.126  13.874  -1.523   0.003   0.718   0.279
E N  4YH7A                279    0.404  59.160   1.234   0.001   0.900   0.099
B Y  4YH7A                280    0.046   9.873   0.594   0.001   0.959   0.040
E T  4YH7A                281    0.240  33.330   1.337   0.001   0.959   0.040
B C  4YH7A                282    0.017   2.331   1.253   0.001   0.959   0.040
E V  4YH7A                283    0.210  32.231   1.321   0.001   0.959   0.040
B A  4YH7A                284    0.021   2.336  -0.078   0.001   0.900   0.099
B M  4YH7A                285    0.272  54.467   0.190   0.001   0.959   0.040
B S  4YH7A                286    0.098  11.462  -0.997   0.003   0.718   0.279
E T  4YH7A                287    0.574  79.558  -1.029   0.004   0.138   0.858
E L  4YH7A                288    0.468  85.746  -1.488   0.004   0.085   0.910
E G  4YH7A                289    0.533  41.923  -2.149   0.004   0.197   0.799
E V  4YH7A                290    0.500  76.865  -1.274   0.004   0.420   0.576
E I  4YH7A                291    0.388  71.799  -1.655   0.003   0.718   0.279
E E  4YH7A                292    0.482  84.223  -0.819   0.002   0.816   0.182
B A  4YH7A                293    0.248  27.319  -0.563   0.002   0.816   0.182
E I  4YH7A                294    0.385  71.244  -0.205   0.002   0.816   0.182
B A  4YH7A                295    0.099  10.899  -0.693   0.002   0.816   0.182
E Q  4YH7A                296    0.354  63.189   0.006   0.002   0.816   0.182
B I  4YH7A                297    0.071  13.154  -0.761   0.002   0.816   0.182
E T  4YH7A                298    0.314  43.607   0.223   0.018   0.846   0.136
B V  4YH7A                299    0.087  13.433  -0.615   0.021   0.756   0.223
E K  4YH7A                300    0.372  76.438   0.014   0.022   0.552   0.426
E A  4YH7A                301    0.370  40.774  -1.188   0.022   0.359   0.619
E L  4YH7A                302    0.379  69.358  -0.892   0.020   0.205   0.775
E P  4YH7A                303    0.422  59.839  -1.419   0.018   0.047   0.935
E K  4YH7A                304    0.582 119.615  -1.697   0.018   0.047   0.935
E P  4YH7A                305    0.385  54.674  -1.101   0.018   0.019   0.964
E P  4YH7A                306    0.389  55.128  -1.562   0.018   0.047   0.935
E G  4YH7A                307    0.518  40.767  -1.384   0.018   0.047   0.935
E T  4YH7A                308    0.597  82.818  -1.044   0.018   0.047   0.935
B P  4YH7A                309    0.276  39.193  -0.976   0.019   0.141   0.840
E V  4YH7A                310    0.536  82.322  -0.286   0.004   0.616   0.381
E V  4YH7A                311    0.375  57.668  -0.394   0.002   0.816   0.182
E T  4YH7A                312    0.306  42.428  -0.264   0.018   0.846   0.136
E E  4YH7A                313    0.568  99.195  -0.568   0.003   0.718   0.279
E S  4YH7A                314    0.346  40.598  -0.099   0.005   0.336   0.660
E T  4YH7A                315    0.546  75.786  -1.294   0.004   0.085   0.910
E A  4YH7A                316    0.553  60.897  -0.482   0.005   0.015   0.979
E T  4YH7A                317    0.543  75.259   0.160   0.005   0.262   0.733
B S  4YH7A                318    0.107  12.564  -0.101   0.003   0.718   0.279
B I  4YH7A                319    0.162  29.914  -0.126   0.001   0.900   0.099
B T  4YH7A                320    0.196  27.241   0.778   0.001   0.959   0.040
B L  4YH7A                321    0.099  18.200   0.629   0.001   0.959   0.040
B T  4YH7A                322    0.080  11.110  -0.634   0.011   0.918   0.071
B W  4YH7A                323    0.167  40.067  -0.359   0.001   0.900   0.099
B D  4YH7A                324    0.226  32.624  -0.661   0.004   0.616   0.381
E S  4YH7A                325    0.377  44.196  -0.333   0.004   0.085   0.910
E G  4YH7A                326    0.633  49.809  -3.144   0.005   0.015   0.979
E N  4YH7A                327    0.652  95.380  -1.527   0.005   0.015   0.979
E P  4YH7A                328    0.377  53.468  -2.313   0.005   0.015   0.979
E E  4YH7A                329    0.502  87.734  -0.980   0.005   0.015   0.979
B P  4YH7A                330    0.213  30.168  -1.142   0.004   0.197   0.799
B V  4YH7A                331    0.224  34.429  -0.201   0.003   0.718   0.279
B S  4YH7A                332    0.137  16.010  -0.291   0.001   0.900   0.099
B Y  4YH7A                333    0.173  36.991   0.352   0.001   0.959   0.040
B Y  4YH7A                334    0.059  12.672   0.642   0.001   0.959   0.040
B I  4YH7A                335    0.130  24.106   0.949   0.000   0.983   0.017
B I  4YH7A                336    0.124  22.903   1.049   0.001   0.959   0.040
B Q  4YH7A                337    0.260  46.365  -0.712   0.002   0.816   0.182
B H  4YH7A                338    0.264  47.949  -0.597   0.023   0.655   0.322
E K  4YH7A                339    0.418  86.003   0.279   0.002   0.816   0.182
E P  4YH7A                340    0.563  79.918  -0.477   0.003   0.718   0.279
E K  4YH7A                341    0.472  97.173  -1.171   0.004   0.138   0.858
E N  4YH7A                342    0.554  81.150  -1.065   0.005   0.045   0.951
E S  4YH7A                343    0.669  78.383  -1.740   0.005   0.015   0.979
E E  4YH7A                344    0.681 119.041  -1.852   0.005   0.045   0.951
E E  4YH7A                345    0.470  82.057  -0.469   0.004   0.197   0.799
E P  4YH7A                346    0.360  51.141  -0.852   0.004   0.514   0.481
B Y  4YH7A                347    0.228  48.745  -0.042   0.002   0.816   0.182
E K  4YH7A                348    0.467  96.000  -0.193   0.001   0.959   0.040
B E  4YH7A                349    0.233  40.653  -0.203   0.001   0.959   0.040
E I  4YH7A                350    0.476  88.042  -0.703   0.001   0.959   0.040
E D  4YH7A                351    0.384  55.349  -0.905   0.002   0.816   0.182
E G  4YH7A                352    0.534  42.018  -1.008   0.005   0.336   0.660
E I  4YH7A                353    0.532  98.346  -1.569   0.005   0.045   0.951
E A  4YH7A                354    0.684  75.399  -0.914   0.004   0.085   0.910
E T  4YH7A                355    0.510  70.682  -0.520   0.005   0.336   0.660
B T  4YH7A                356    0.177  24.619  -0.696   0.003   0.718   0.279
E R  4YH7A                357    0.346  79.303   0.652   0.001   0.900   0.099
B Y  4YH7A                358    0.056  11.924   0.187   0.001   0.900   0.099
E S  4YH7A                359    0.375  43.985   1.090   0.001   0.900   0.099
B V  4YH7A                360    0.053   8.161  -0.297   0.001   0.900   0.099
E A  4YH7A                361    0.450  49.601   0.361   0.004   0.616   0.381
E G  4YH7A                362    0.554  43.623  -0.352   0.005   0.336   0.660
B L  4YH7A                363    0.068  12.432  -0.543   0.004   0.138   0.858
E S  4YH7A                364    0.498  58.319   0.713   0.004   0.138   0.858
E P  4YH7A                365    0.495  70.184  -0.196   0.004   0.138   0.858
E Y  4YH7A                366    0.547 116.915  -1.279   0.005   0.045   0.951
B S  4YH7A                367    0.245  28.726  -1.213   0.004   0.138   0.858
E D  4YH7A                368    0.463  66.689   0.188   0.004   0.616   0.381
B Y  4YH7A                369    0.071  15.151  -0.558   0.001   0.900   0.099
E E  4YH7A                370    0.328  57.302   1.778   0.001   0.959   0.040
B F  4YH7A                371    0.027   5.359   0.723   0.001   0.959   0.040
B R  4YH7A                372    0.159  36.411   1.509   0.001   0.959   0.040
B V  4YH7A                373    0.012   1.798   1.793   0.001   0.959   0.040
B V  4YH7A                374    0.163  25.068   1.890   0.006   0.962   0.032
B A  4YH7A                375    0.016   1.818   0.715   0.001   0.959   0.040
B V  4YH7A                376    0.178  27.374   0.392   0.001   0.959   0.040
B N  4YH7A                377    0.109  16.016  -0.331   0.003   0.718   0.279
E N  4YH7A                378    0.543  79.524  -0.574   0.019   0.141   0.840
E I  4YH7A                379    0.460  85.155  -1.312   0.004   0.085   0.910
E G  4YH7A                380    0.381  29.993  -2.166   0.005   0.262   0.733
E R  4YH7A                381    0.551 126.293  -1.229   0.005   0.262   0.733
B G  4YH7A                382    0.304  23.893  -1.814   0.005   0.262   0.733
E P  4YH7A                383    0.557  78.996  -0.437   0.005   0.336   0.660
B A  4YH7A                384    0.302  33.280  -1.019   0.005   0.262   0.733
E S  4YH7A                385    0.339  39.707  -1.443   0.005   0.336   0.660
B E  4YH7A                386    0.280  48.881  -0.623   0.004   0.616   0.381
E P  4YH7A                387    0.335  47.593  -0.028   0.002   0.816   0.182
B V  4YH7A                388    0.105  16.062  -0.212   0.002   0.816   0.182
E L  4YH7A                389    0.343  62.730   0.403   0.011   0.918   0.071
B T  4YH7A                390    0.120  16.616  -0.340   0.011   0.918   0.071
E Q  4YH7A                391    0.290  51.865   0.429   0.018   0.846   0.136
B T  4YH7A                392    0.295  40.944  -0.321   0.023   0.655   0.322
E S  4YH7A                393    0.423  49.540  -0.598   0.021   0.279   0.699
E E  4YH7A                394    0.506  88.346  -0.870   0.018   0.088   0.893
E Q  4YH7A                395    0.588 105.053  -1.382   0.018   0.088   0.893
E A  4YH7A                396    0.504  55.552  -0.853   0.018   0.088   0.893
E P  4YH7A                397    0.285  40.427  -0.774   0.018   0.047   0.935
E S  4YH7A                398    0.535  62.761  -1.896   0.018   0.047   0.935
E S  4YH7A                399    0.517  60.616  -1.551   0.018   0.019   0.964
E A  4YH7A                400    0.378  41.711  -0.535   0.018   0.019   0.964
B P  4YH7A                401    0.254  36.014  -1.701   0.018   0.019   0.964
E R  4YH7A                402    0.570 130.530  -0.785   0.018   0.088   0.893
E D  4YH7A                403    0.455  65.537  -0.866   0.004   0.197   0.799
B V  4YH7A                404    0.136  20.842  -0.135   0.004   0.514   0.481
E Q  4YH7A                405    0.551  98.355   0.353   0.002   0.816   0.182
B A  4YH7A                406    0.158  17.401  -0.874   0.001   0.900   0.099
E R  4YH7A                407    0.441 100.989  -0.469   0.001   0.900   0.099
E M  4YH7A                408    0.413  82.701  -1.390   0.003   0.718   0.279
E L  4YH7A                409    0.419  76.719  -1.094   0.004   0.514   0.481
E S  4YH7A                410    0.505  59.209  -0.827   0.004   0.197   0.799
E S  4YH7A                411    0.576  67.519  -0.564   0.004   0.085   0.910
E T  4YH7A                412    0.392  54.357  -0.520   0.005   0.336   0.660
E T  4YH7A                413    0.320  44.439   0.670   0.001   0.900   0.099
B I  4YH7A                414    0.028   5.235   0.158   0.001   0.959   0.040
E L  4YH7A                415    0.291  53.264   1.604   0.006   0.962   0.032
B V  4YH7A                416    0.024   3.643   1.055   0.006   0.962   0.032
E Q  4YH7A                417    0.250  44.614   0.998   0.011   0.918   0.071
B W  4YH7A                418    0.038   9.259  -0.360   0.001   0.900   0.099
E K  4YH7A                419    0.470  96.741   1.132   0.001   0.900   0.099
B E  4YH7A                420    0.159  27.690  -0.598   0.004   0.514   0.481
E P  4YH7A                421    0.359  50.999  -0.388   0.004   0.138   0.858
E E  4YH7A                422    0.593 103.615  -2.738   0.005   0.045   0.951
E E  4YH7A                423    0.557  97.360  -0.912   0.005   0.045   0.951
E P  4YH7A                424    0.600  85.069  -2.270   0.005   0.015   0.979
E N  4YH7A                425    0.626  91.632  -2.153   0.005   0.015   0.979
B G  4YH7A                426    0.187  14.693  -2.733   0.005   0.045   0.951
E Q  4YH7A                427    0.417  74.440   0.451   0.005   0.336   0.660
B I  4YH7A                428    0.058  10.748  -1.018   0.003   0.718   0.279
E Q  4YH7A                429    0.257  45.972   0.325   0.002   0.816   0.182
B G  4YH7A                430    0.130  10.262  -0.723   0.001   0.900   0.099
B Y  4YH7A                431    0.062  13.185   0.271   0.001   0.900   0.099
B R  4YH7A                432    0.179  40.991   0.843   0.001   0.959   0.040
B V  4YH7A                433    0.052   8.008  -0.404   0.001   0.959   0.040
B Y  4YH7A                434    0.167  35.688  -0.675   0.001   0.959   0.040
B Y  4YH7A                435    0.108  23.101  -0.179   0.001   0.959   0.040
E T  4YH7A                436    0.300  41.610   0.269   0.001   0.900   0.099
E M  4YH7A                437    0.359  71.836  -0.605   0.003   0.718   0.279
E D  4YH7A                438    0.478  68.909  -0.647   0.004   0.197   0.799
E P  4YH7A                439    0.602  85.410  -2.420   0.005   0.045   0.951
E T  4YH7A                440    0.575  79.808  -2.118   0.018   0.047   0.935
E Q  4YH7A                441    0.600 107.106  -1.920   0.018   0.088   0.893
E H  4YH7A                442    0.542  98.608  -0.824   0.019   0.141   0.840
E V  4YH7A                443    0.349  53.687  -1.314   0.005   0.262   0.733
E N  4YH7A                444    0.424  62.132  -0.927   0.004   0.514   0.481
E N  4YH7A                445    0.402  58.838  -0.429   0.003   0.718   0.279
B W  4YH7A                446    0.278  66.979  -0.417   0.002   0.816   0.182
E M  4YH7A                447    0.346  69.295  -0.620   0.001   0.959   0.040
E K  4YH7A                448    0.473  97.234  -0.396   0.001   0.959   0.040
E H  4YH7A                449    0.356  64.684   0.061   0.001   0.959   0.040
E N  4YH7A                450    0.431  63.098  -0.478   0.001   0.900   0.099
E V  4YH7A                451    0.394  60.527  -0.708   0.004   0.420   0.576
E A  4YH7A                452    0.563  61.999  -1.626   0.005   0.015   0.979
E D  4YH7A                453    0.721 103.882  -0.601   0.005   0.015   0.979
E S  4YH7A                454    0.453  53.138  -0.243   0.005   0.336   0.660
B Q  4YH7A                455    0.187  33.362   0.148   0.003   0.718   0.279
E I  4YH7A                456    0.271  50.190   1.070   0.001   0.900   0.099
B T  4YH7A                457    0.048   6.699   1.012   0.001   0.959   0.040
E T  4YH7A                458    0.283  39.210   1.738   0.001   0.959   0.040
B I  4YH7A                459    0.032   5.920  -0.064   0.001   0.900   0.099
E G  4YH7A                460    0.502  39.484   0.914   0.004   0.616   0.381
E N  4YH7A                461    0.594  86.991   0.112   0.005   0.336   0.660
B L  4YH7A                462    0.077  14.007   0.307   0.004   0.138   0.858
E V  4YH7A                463    0.481  73.960   1.212   0.004   0.197   0.799
E P  4YH7A                464    0.511  72.511   0.375   0.004   0.197   0.799
E Q  4YH7A                465    0.535  95.587  -0.742   0.005   0.045   0.951
E K  4YH7A                466    0.279  57.308   0.221   0.004   0.197   0.799
E T  4YH7A                467    0.448  62.138   1.085   0.003   0.718   0.279
B Y  4YH7A                468    0.069  14.852   0.085   0.001   0.959   0.040
E S  4YH7A                469    0.310  36.297   2.096   0.001   0.959   0.040
B V  4YH7A                470    0.024   3.689   1.165   0.001   0.959   0.040
B K  4YH7A                471    0.158  32.418   2.071   0.001   0.959   0.040
B V  4YH7A                472    0.011   1.706   1.904   0.001   0.959   0.040
B L  4YH7A                473    0.138  25.286   1.673   0.006   0.962   0.032
B A  4YH7A                474    0.016   1.774   0.769   0.001   0.959   0.040
B F  4YH7A                475    0.156  31.269   0.481   0.001   0.959   0.040
B T  4YH7A                476    0.082  11.401  -0.394   0.003   0.718   0.279
E S  4YH7A                477    0.523  61.260  -0.618   0.004   0.138   0.858
E I  4YH7A                478    0.426  78.829  -1.131   0.004   0.085   0.910
E G  4YH7A                479    0.358  28.167  -2.236   0.005   0.336   0.660
E D  4YH7A                480    0.560  80.725  -0.915   0.005   0.262   0.733
B G  4YH7A                481    0.246  19.352  -1.609   0.004   0.197   0.799
E P  4YH7A                482    0.621  88.134  -0.275   0.005   0.262   0.733
E L  4YH7A                483    0.400  73.258  -0.561   0.005   0.262   0.733
B S  4YH7A                484    0.273  32.007  -1.087   0.005   0.262   0.733
E S  4YH7A                485    0.330  38.641  -1.002   0.004   0.514   0.481
E D  4YH7A                486    0.357  51.458   0.183   0.002   0.816   0.182
B I  4YH7A                487    0.059  10.933   0.365   0.001   0.900   0.099
E Q  4YH7A                488    0.319  56.991   0.867   0.001   0.959   0.040
B V  4YH7A                489    0.058   8.945  -0.522   0.001   0.900   0.099
E I  4YH7A                490    0.299  55.352   0.844   0.002   0.816   0.182
B T  4YH7A                491    0.229  31.748  -0.601   0.021   0.451   0.528
E Q  4YH7A                492    0.399  71.351  -0.281   0.019   0.141   0.840
E T  4YH7A                493    0.538  74.662  -0.954   0.018   0.047   0.935
E G  4YH7A                494    0.638  50.226  -1.146   0.018   0.047   0.935
E V  4YH7A                495    0.374  57.499  -0.287   0.018   0.047   0.935
B P  4YH7A                496    0.252  35.830  -1.415   0.018   0.019   0.964
E G  4YH7A                497    0.592  46.551  -1.102   0.018   0.019   0.964
E Q  4YH7A                498    0.465  82.978   0.165   0.018   0.019   0.964
B P  4YH7A                499    0.122  17.354  -1.621   0.018   0.019   0.964
E L  4YH7A                500    0.536  98.160  -0.588   0.004   0.085   0.910
E N  4YH7A                501    0.568  83.155   0.162   0.004   0.197   0.799
B F  4YH7A                502    0.103  20.692   0.142   0.004   0.514   0.481
E K  4YH7A                503    0.512 105.236   0.982   0.001   0.900   0.099
B A  4YH7A                504    0.117  12.882  -0.372   0.001   0.959   0.040
E E  4YH7A                505    0.429  74.859   0.502   0.001   0.959   0.040
E P  4YH7A                506    0.432  61.343  -1.060   0.001   0.900   0.099
E E  4YH7A                507    0.407  71.016  -0.792   0.004   0.616   0.381
E S  4YH7A                508    0.453  53.127  -1.364   0.004   0.138   0.858
E E  4YH7A                509    0.631 110.218  -0.602   0.005   0.045   0.951
E T  4YH7A                510    0.450  62.360  -0.288   0.004   0.197   0.799
E S  4YH7A                511    0.333  39.051   0.499   0.002   0.816   0.182
B I  4YH7A                512    0.037   6.900  -0.088   0.001   0.900   0.099
E L  4YH7A                513    0.279  51.085   1.624   0.001   0.959   0.040
B L  4YH7A                514    0.023   4.230   0.896   0.001   0.959   0.040
E S  4YH7A                515    0.270  31.632   1.285   0.001   0.959   0.040
B W  4YH7A                516    0.034   8.105  -0.527   0.001   0.900   0.099
E T  4YH7A                517    0.416  57.727   1.006   0.001   0.900   0.099
E P  4YH7A                518    0.280  39.718  -0.212   0.004   0.514   0.481
E P  4YH7A                519    0.272  38.568  -0.043   0.004   0.085   0.910
E R  4YH7A                520    0.650 148.964  -1.915   0.005   0.015   0.979
E S  4YH7A                521    0.750  87.900  -1.481   0.005   0.015   0.979
E D  4YH7A                522    0.624  89.947  -2.524   0.003   0.003   0.994
E T  4YH7A                523    0.551  76.382  -0.385   0.005   0.045   0.951
B I  4YH7A                524    0.101  18.722  -1.192   0.005   0.262   0.733
E A  4YH7A                525    0.374  41.171   0.600   0.004   0.514   0.481
B S  4YH7A                526    0.242  28.398  -0.150   0.002   0.816   0.182
B Y  4YH7A                527    0.054  11.561   0.824   0.001   0.900   0.099
B E  4YH7A                528    0.170  29.647   0.781   0.001   0.959   0.040
B L  4YH7A                529    0.037   6.775  -0.434   0.001   0.959   0.040
B V  4YH7A                530    0.163  25.038   0.297   0.001   0.959   0.040
B Y  4YH7A                531    0.071  15.109  -0.112   0.001   0.959   0.040
E R  4YH7A                532    0.261  59.861   0.522   0.001   0.959   0.040
E D  4YH7A                533    0.483  69.644  -0.256   0.003   0.718   0.279
E G  4YH7A                534    0.542  42.687  -1.022   0.004   0.085   0.910
E D  4YH7A                535    0.715 103.032  -1.759   0.005   0.015   0.979
E Q  4YH7A                536    0.653 116.715  -1.279   0.005   0.015   0.979
E G  4YH7A                537    0.570  44.851  -1.366   0.005   0.045   0.951
E E  4YH7A                538    0.520  90.931  -0.128   0.004   0.197   0.799
E E  4YH7A                539    0.297  51.903  -0.291   0.004   0.514   0.481
E Q  4YH7A                540    0.447  79.924  -0.227   0.003   0.718   0.279
E R  4YH7A                541    0.456 104.470   0.210   0.001   0.900   0.099
B I  4YH7A                542    0.221  40.830  -0.367   0.001   0.959   0.040
E T  4YH7A                543    0.448  62.165  -0.780   0.001   0.959   0.040
E I  4YH7A                544    0.377  69.838  -0.485   0.003   0.718   0.279
E E  4YH7A                545    0.368  64.237  -0.598   0.004   0.138   0.858
E P  4YH7A                546    0.711 100.891  -0.676   0.005   0.015   0.979
E G  4YH7A                547    0.587  46.158  -0.177   0.004   0.085   0.910
B T  4YH7A                548    0.179  24.813  -0.149   0.004   0.514   0.481
E S  4YH7A                549    0.332  38.875   1.000   0.001   0.900   0.099
B Y  4YH7A                550    0.059  12.566   1.002   0.001   0.959   0.040
E R  4YH7A                551    0.278  63.616   1.934   0.001   0.959   0.040
B L  4YH7A                552    0.035   6.354   0.469   0.001   0.959   0.040
E Q  4YH7A                553    0.485  86.550   1.538   0.003   0.718   0.279
E G  4YH7A                554    0.516  40.586  -0.053   0.004   0.420   0.576
B L  4YH7A                555    0.080  14.685   0.451   0.004   0.197   0.799
E K  4YH7A                556    0.482  99.230   1.241   0.004   0.197   0.799
E P  4YH7A                557    0.489  69.347  -0.004   0.004   0.197   0.799
E N  4YH7A                558    0.588  86.025  -1.329   0.005   0.045   0.951
B S  4YH7A                559    0.243  28.480  -0.446   0.004   0.138   0.858
E L  4YH7A                560    0.439  80.381   1.076   0.003   0.718   0.279
B Y  4YH7A                561    0.071  15.215   0.093   0.001   0.959   0.040
E Y  4YH7A                562    0.287  61.225   2.282   0.001   0.959   0.040
B F  4YH7A                563    0.022   4.435   1.274   0.001   0.959   0.040
B R  4YH7A                564    0.127  29.083   1.782   0.001   0.959   0.040
B L  4YH7A                565    0.011   1.923   1.975   0.001   0.959   0.040
B S  4YH7A                566    0.134  15.658   1.747   0.006   0.962   0.032
B A  4YH7A                567    0.015   1.708   1.034   0.001   0.959   0.040
B R  4YH7A                568    0.160  36.571   0.666   0.001   0.959   0.040
B S  4YH7A                569    0.069   8.028  -0.134   0.003   0.718   0.279
E P  4YH7A                570    0.493  69.914  -0.601   0.004   0.085   0.910
E Q  4YH7A                571    0.463  82.674  -0.782   0.004   0.085   0.910
E G  4YH7A                572    0.326  25.625  -2.252   0.005   0.336   0.660
E L  4YH7A                573    0.559 102.316  -1.143   0.005   0.262   0.733
B G  4YH7A                574    0.234  18.416  -1.271   0.004   0.197   0.799
E A  4YH7A                575    0.609  67.112  -0.187   0.004   0.197   0.799
E S  4YH7A                576    0.386  45.227  -0.468   0.005   0.262   0.733
B T  4YH7A                577    0.257  35.618  -1.274   0.005   0.262   0.733
E A  4YH7A                578    0.318  35.022  -0.783   0.004   0.514   0.481
E E  4YH7A                579    0.406  70.841   0.534   0.002   0.816   0.182
B I  4YH7A                580    0.064  11.914   0.187   0.001   0.900   0.099
E S  4YH7A                581    0.405  47.443   1.020   0.001   0.900   0.099
B A  4YH7A                582    0.066   7.262  -0.078   0.001   0.900   0.099
E R  4YH7A                583    0.374  85.577   0.945   0.001   0.900   0.099
B T  4YH7A                584    0.145  20.056  -0.071   0.003   0.718   0.279
E M  4YH7A                585    0.414  82.881   0.105   0.021   0.279   0.699
E Q  4YH7A                586    0.593 105.856  -0.861   0.018   0.047   0.935
E S  4YH7A                587    0.676  79.204  -1.116   0.018   0.047   0.935
E M  4YH7A                588    0.331  66.193  -1.319   0.018   0.047   0.935
E F  4YH7A                589    0.313  62.819  -0.935   0.018   0.019   0.964
E A  4YH7A                590    0.390  42.978  -1.545   0.018   0.047   0.935
E K  4YH7A                591    0.603 123.934  -0.567   0.004   0.085   0.910
E N  4YH7A                592    0.452  66.173  -1.003   0.004   0.138   0.858
B F  4YH7A                593    0.145  29.041  -0.173   0.004   0.420   0.576
E H  4YH7A                594    0.576 104.756   0.684   0.002   0.816   0.182
B V  4YH7A                595    0.111  17.061  -0.666   0.001   0.900   0.099
E K  4YH7A                596    0.516 106.059   0.140   0.001   0.959   0.040
E A  4YH7A                597    0.377  41.501  -0.902   0.002   0.816   0.182
E V  4YH7A                598    0.365  56.131  -0.472   0.004   0.616   0.381
E M  4YH7A                599    0.470  94.107  -0.779   0.004   0.085   0.910
E K  4YH7A                600    0.594 122.124  -0.577   0.005   0.015   0.979
E T  4YH7A                601    0.451  62.512  -0.188   0.005   0.262   0.733
E S  4YH7A                602    0.274  32.160   0.575   0.002   0.816   0.182
B V  4YH7A                603    0.031   4.795  -0.043   0.001   0.900   0.099
E L  4YH7A                604    0.284  51.927   1.509   0.006   0.962   0.032
B L  4YH7A                605    0.028   5.072   0.683   0.001   0.959   0.040
E S  4YH7A                606    0.288  33.789   1.165   0.001   0.900   0.099
B W  4YH7A                607    0.045  10.847  -0.059   0.001   0.900   0.099
E E  4YH7A                608    0.509  88.922   1.567   0.001   0.900   0.099
E I  4YH7A                609    0.258  47.822   0.134   0.004   0.616   0.381
E P  4YH7A                610    0.367  52.120  -0.296   0.004   0.197   0.799
E E  4YH7A                611    0.640 111.878  -2.137   0.005   0.045   0.951
E N  4YH7A                612    0.515  75.352  -0.547   0.005   0.045   0.951
E Y  4YH7A                613    0.664 141.833  -2.552   0.005   0.045   0.951
E N  4YH7A                614    0.553  80.915  -2.070   0.005   0.045   0.951
B S  4YH7A                615    0.259  30.378  -0.941   0.004   0.197   0.799
E A  4YH7A                616    0.291  32.035  -0.326   0.004   0.616   0.381
B M  4YH7A                617    0.114  22.851   0.189   0.001   0.900   0.099
B P  4YH7A                618    0.166  23.584  -0.168   0.001   0.959   0.040
B F  4YH7A                619    0.050  10.075   0.619   0.001   0.959   0.040
B K  4YH7A                620    0.180  36.985   1.100   0.001   0.959   0.040
B I  4YH7A                621    0.052   9.638   0.628   0.001   0.959   0.040
B L  4YH7A                622    0.222  40.721   0.237   0.001   0.959   0.040
B Y  4YH7A                623    0.149  31.756   0.228   0.001   0.959   0.040
E D  4YH7A                624    0.453  65.292   0.582   0.004   0.616   0.381
E D  4YH7A                625    0.788 113.493  -0.610   0.004   0.085   0.910
E G  4YH7A                626    0.672  52.902  -1.745   0.004   0.085   0.910
E K  4YH7A                627    0.627 128.933  -0.427   0.004   0.420   0.576
E M  4YH7A                628    0.404  80.760   0.217   0.003   0.718   0.279
E V  4YH7A                629    0.403  61.941  -0.733   0.002   0.816   0.182
E E  4YH7A                630    0.415  72.501  -0.566   0.001   0.900   0.099
E E  4YH7A                631    0.465  81.323  -0.297   0.001   0.900   0.099
B V  4YH7A                632    0.222  34.152  -0.172   0.001   0.900   0.099
E D  4YH7A                633    0.544  78.390  -0.295   0.004   0.514   0.481
E G  4YH7A                634    0.422  33.219  -1.404   0.005   0.045   0.951
E R  4YH7A                635    0.665 152.377  -0.408   0.005   0.045   0.951
E A  4YH7A                636    0.440  48.499   0.157   0.004   0.420   0.576
B T  4YH7A                637    0.173  23.995   0.162   0.002   0.816   0.182
E Q  4YH7A                638    0.315  56.188   0.946   0.001   0.900   0.099
B K  4YH7A                639    0.056  11.540   1.090   0.001   0.959   0.040
E L  4YH7A                640    0.271  49.657   1.658   0.001   0.959   0.040
B I  4YH7A                641    0.032   5.883   0.333   0.001   0.900   0.099
E V  4YH7A                642    0.484  74.345   1.243   0.003   0.718   0.279
E N  4YH7A                643    0.475  69.584   0.060   0.004   0.420   0.576
B L  4YH7A                644    0.081  14.776   0.333   0.004   0.138   0.858
E K  4YH7A                645    0.497 102.253   1.169   0.004   0.138   0.858
E P  4YH7A                646    0.529  75.093   0.335   0.004   0.138   0.858
E E  4YH7A                647    0.597 104.243  -1.080   0.005   0.045   0.951
E K  4YH7A                648    0.250  51.466  -0.419   0.004   0.138   0.858
E S  4YH7A                649    0.391  45.884   1.221   0.003   0.718   0.279
B Y  4YH7A                650    0.055  11.668  -0.082   0.001   0.959   0.040
E S  4YH7A                651    0.266  31.187   2.326   0.001   0.959   0.040
B F  4YH7A                652    0.020   3.994   1.432   0.001   0.959   0.040
B V  4YH7A                653    0.140  21.503   1.850   0.001   0.959   0.040
B L  4YH7A                654    0.010   1.849   1.946   0.001   0.959   0.040
B T  4YH7A                655    0.154  21.388   2.128   0.001   0.959   0.040
B N  4YH7A                656    0.017   2.460   0.612   0.001   0.959   0.040
B R  4YH7A                657    0.218  50.014   0.689   0.001   0.959   0.040
B G  4YH7A                658    0.092   7.201  -0.317   0.004   0.616   0.381
E N  4YH7A                659    0.545  79.817  -0.704   0.004   0.138   0.858
E S  4YH7A                660    0.516  60.487  -0.970   0.004   0.085   0.910
E A  4YH7A                661    0.356  39.198  -2.396   0.004   0.138   0.858
E G  4YH7A                662    0.604  47.558  -1.820   0.005   0.045   0.951
E G  4YH7A                663    0.374  29.434  -1.793   0.004   0.085   0.910
E L  4YH7A                664    0.500  91.605  -0.409   0.005   0.336   0.660
E Q  4YH7A                665    0.417  74.423  -0.880   0.004   0.514   0.481
B H  4YH7A                666    0.280  50.950  -0.522   0.003   0.718   0.279
E R  4YH7A                667    0.475 108.821   0.052   0.002   0.816   0.182
B V  4YH7A                668    0.144  22.164   0.366   0.001   0.900   0.099
E T  4YH7A                669    0.404  56.021   0.474   0.001   0.959   0.040
B A  4YH7A                670    0.078   8.540  -0.609   0.001   0.900   0.099
E K  4YH7A                671    0.372  76.582   0.502   0.002   0.816   0.182
B T  4YH7A                672    0.106  14.633  -0.513   0.022   0.552   0.426
E A  4YH7A                673    0.399  43.915   0.269   0.019   0.141   0.840
E P  4YH7A                674    0.466  66.097  -0.685   0.018   0.047   0.935
E D  4YH7A                675    0.688  99.112  -1.312   0.018   0.047   0.935
E V  4YH7A                676    0.411  63.109  -0.986   0.018   0.047   0.935
B L  4YH7A                677    0.258  47.313  -1.356   0.018   0.047   0.935
E R  4YH7A                678    0.634 145.117  -1.252   0.018   0.019   0.964
E T  4YH7A                679    0.498  69.073  -1.467   0.018   0.019   0.964
E K  4YH7A                680    0.368  75.615  -0.719   0.018   0.019   0.964
E P  4YH7A                681    0.319  45.266  -1.605   0.018   0.047   0.935
E A  4YH7A                682    0.514  56.665  -0.837   0.020   0.205   0.775
E F  4YH7A                683    0.341  68.378  -1.076   0.021   0.451   0.528
B I  4YH7A                684    0.235  43.568  -0.403   0.021   0.756   0.223
E G  4YH7A                685    0.534  41.994  -0.608   0.018   0.846   0.136
B K  4YH7A                686    0.278  57.246  -0.415   0.018   0.846   0.136
E T  4YH7A                687    0.459  63.608  -0.369   0.004   0.616   0.381
E N  4YH7A                688    0.489  71.516  -1.533   0.004   0.197   0.799
E L  4YH7A                689    0.452  82.743  -1.199   0.005   0.045   0.951
E D  4YH7A                690    0.615  88.679  -1.189   0.005   0.045   0.951
B G  4YH7A                691    0.167  13.135  -0.918   0.005   0.336   0.660
B M  4YH7A                692    0.183  36.578  -0.277   0.002   0.816   0.182
B I  4YH7A                693    0.067  12.358   0.567   0.001   0.959   0.040
B T  4YH7A                694    0.158  21.915   0.350   0.001   0.959   0.040
B V  4YH7A                695    0.069  10.682   0.064   0.001   0.959   0.040
B Q  4YH7A                696    0.229  40.846  -0.324   0.001   0.900   0.099
B L  4YH7A                697    0.150  27.465   0.086   0.004   0.616   0.381
E P  4YH7A                698    0.370  52.574  -0.296   0.005   0.262   0.733
E D  4YH7A                699    0.525  75.580  -1.577   0.004   0.138   0.858
E V  4YH7A                700    0.346  53.180  -0.948   0.005   0.045   0.951
E P  4YH7A                701    0.578  81.961  -1.285   0.003   0.003   0.994
E A  4YH7A                702    0.617  68.004  -2.259   0.003   0.003   0.994
E N  4YH7A                703    0.705 103.197  -2.300   0.003   0.003   0.994
E E  4YH7A                704    0.331  57.791  -2.347   0.005   0.015   0.979
E N  4YH7A                705    0.465  68.017  -0.136   0.004   0.085   0.910
B I  4YH7A                706    0.071  13.135  -0.465   0.004   0.420   0.576
E K  4YH7A                707    0.282  57.966   0.278   0.004   0.616   0.381
B G  4YH7A                708    0.216  16.968  -0.281   0.002   0.816   0.182
B Y  4YH7A                709    0.038   8.142   0.201   0.001   0.900   0.099
B Y  4YH7A                710    0.160  34.107   1.287   0.001   0.959   0.040
B I  4YH7A                711    0.033   6.123   0.229   0.001   0.959   0.040
B I  4YH7A                712    0.142  26.251   0.821   0.001   0.959   0.040
B I  4YH7A                713    0.075  13.912   0.197   0.001   0.959   0.040
E V  4YH7A                714    0.250  38.425   1.398   0.001   0.959   0.040
E P  4YH7A                715    0.471  66.807  -0.135   0.001   0.900   0.099
E L  4YH7A                716    0.365  66.868  -0.267   0.005   0.336   0.660
E K  4YH7A                717    0.663 136.461  -1.085   0.004   0.085   0.910
E K  4YH7A                718    0.676 138.991  -0.381   0.005   0.015   0.979
E S  4YH7A                719    0.666  78.043  -1.348   0.004   0.085   0.910
E R  4YH7A                720    0.605 138.522  -0.284   0.004   0.197   0.799
E G  4YH7A                721    0.333  26.183  -0.922   0.004   0.420   0.576
E K  4YH7A                722    0.368  75.780   0.022   0.002   0.816   0.182
E F  4YH7A                723    0.401  80.461  -0.376   0.001   0.959   0.040
B I  4YH7A                724    0.203  37.463  -0.103   0.001   0.959   0.040
E K  4YH7A                725    0.434  89.233  -0.571   0.001   0.900   0.099
B P  4YH7A                726    0.220  31.147  -0.676   0.003   0.718   0.279
E W  4YH7A                727    0.524 125.950  -1.511   0.005   0.262   0.733
E E  4YH7A                728    0.548  95.701  -1.540   0.005   0.045   0.951
E S  4YH7A                729    0.539  63.136  -1.087   0.005   0.045   0.951
E P  4YH7A                730    0.408  57.966  -0.923   0.004   0.138   0.858
E D  4YH7A                731    0.417  60.032  -0.464   0.004   0.420   0.576
E E  4YH7A                732    0.358  62.595   0.295   0.002   0.816   0.182
B M  4YH7A                733    0.095  18.909   0.257   0.001   0.900   0.099
E E  4YH7A                734    0.419  73.182   0.712   0.001   0.900   0.099
B L  4YH7A                735    0.091  16.625  -0.099   0.018   0.846   0.136
E D  4YH7A                736    0.551  79.327   0.289   0.021   0.451   0.528
E E  4YH7A                737    0.610 106.602  -1.131   0.004   0.138   0.858
B L  4YH7A                738    0.190  34.881  -0.571   0.004   0.138   0.858
E L  4YH7A                739    0.513  93.985  -0.546   0.004   0.138   0.858
E K  4YH7A                740    0.626 128.748  -0.775   0.004   0.085   0.910
E E  4YH7A                741    0.572  99.893  -2.221   0.005   0.045   0.951
B I  4YH7A                742    0.255  47.138  -0.883   0.005   0.336   0.660
E S  4YH7A                743    0.356  41.688   0.504   0.002   0.816   0.182
B R  4YH7A                744    0.088  20.175   0.194   0.001   0.959   0.040
B K  4YH7A                745    0.214  44.102   0.624   0.001   0.959   0.040
B R  4YH7A                746    0.046  10.580   0.743   0.001   0.959   0.040
B R  4YH7A                747    0.105  23.931   0.493   0.001   0.959   0.040
B S  4YH7A                748    0.032   3.739  -0.080   0.001   0.959   0.040
B I  4YH7A                749    0.099  18.315   0.599   0.001   0.959   0.040
B R  4YH7A                750    0.068  15.572  -1.815   0.001   0.959   0.040
B Y  4YH7A                751    0.165  35.175  -0.552   0.001   0.900   0.099
E G  4YH7A                752    0.271  21.343  -0.048   0.004   0.420   0.576
E R  4YH7A                753    0.508 116.355  -1.358   0.004   0.197   0.799
E E  4YH7A                754    0.660 115.319  -1.597   0.004   0.197   0.799
E V  4YH7A                755    0.431  66.306  -1.919   0.004   0.197   0.799
E E  4YH7A                756    0.607 106.078  -1.819   0.005   0.262   0.733
E L  4YH7A                757    0.376  68.882  -1.889   0.005   0.336   0.660
E K  4YH7A                758    0.486  99.991  -0.940   0.005   0.336   0.660
E P  4YH7A                759    0.341  48.416  -0.883   0.004   0.420   0.576
B Y  4YH7A                760    0.246  52.634  -0.618   0.003   0.718   0.279
B I  4YH7A                761    0.232  42.846  -0.446   0.002   0.816   0.182
E A  4YH7A                762    0.331  36.531  -0.584   0.003   0.718   0.279
B A  4YH7A                763    0.211  23.274  -1.577   0.023   0.655   0.322
E H  4YH7A                764    0.473  86.039  -0.665   0.023   0.655   0.322
B F  4YH7A                765    0.264  53.045  -1.503   0.021   0.451   0.528
E D  4YH7A                766    0.543  78.290  -1.557   0.020   0.205   0.775
E V  4YH7A                767    0.547  84.135  -1.363   0.019   0.141   0.840
E L  4YH7A                768    0.269  49.254  -0.274   0.018   0.047   0.935
E P  4YH7A                769    0.435  61.698  -0.381   0.018   0.019   0.964
E T  4YH7A                770    0.491  68.157  -1.074   0.019   0.141   0.840
E E  4YH7A                771    0.523  91.386  -0.783   0.022   0.359   0.619
B F  4YH7A                772    0.164  32.814  -0.766   0.023   0.655   0.322
E T  4YH7A                773    0.376  52.207  -0.254   0.023   0.655   0.322
B L  4YH7A                774    0.175  32.042  -1.237   0.022   0.552   0.426
E G  4YH7A                775    0.339  26.719  -1.653   0.021   0.279   0.699
E D  4YH7A                776    0.492  70.854  -1.459   0.020   0.205   0.775
E D  4YH7A                777    0.570  82.166  -1.439   0.018   0.088   0.893
E K  4YH7A                778    0.504 103.673  -1.166   0.004   0.085   0.910
E H  4YH7A                779    0.545  99.136  -0.849   0.005   0.262   0.733
E Y  4YH7A                780    0.280  59.879  -0.036   0.005   0.336   0.660
E G  4YH7A                781    0.387  30.481  -0.421   0.005   0.336   0.660
E G  4YH7A                782    0.334  26.302  -0.732   0.003   0.718   0.279
B F  4YH7A                783    0.128  25.750  -0.000   0.001   0.900   0.099
E T  4YH7A                784    0.411  56.950   0.853   0.001   0.900   0.099
B N  4YH7A                785    0.156  22.868  -0.029   0.003   0.718   0.279
E K  4YH7A                786    0.550 113.114   0.615   0.005   0.262   0.733
E Q  4YH7A                787    0.597 106.606   0.361   0.004   0.085   0.910
B L  4YH7A                788    0.069  12.689  -0.452   0.004   0.085   0.910
E Q  4YH7A                789    0.474  84.585   0.882   0.004   0.085   0.910
E S  4YH7A                790    0.532  62.350  -0.435   0.005   0.045   0.951
E G  4YH7A                791    0.577  45.426  -0.976   0.005   0.045   0.951
B Q  4YH7A                792    0.196  34.952   0.314   0.005   0.262   0.733
E E  4YH7A                793    0.409  71.470   1.531   0.001   0.900   0.099
B Y  4YH7A                794    0.044   9.403   0.877   0.001   0.959   0.040
B V  4YH7A                795    0.241  36.965   2.214   0.001   0.959   0.040
B F  4YH7A                796    0.019   3.894   1.448   0.001   0.959   0.040
B F  4YH7A                797    0.096  19.267   1.490   0.001   0.959   0.040
B V  4YH7A                798    0.015   2.275   1.653   0.006   0.962   0.032
B L  4YH7A                799    0.139  25.433   1.776   0.001   0.959   0.040
B A  4YH7A                800    0.017   1.873   0.660   0.001   0.959   0.040
B V  4YH7A                801    0.120  18.367   0.350   0.001   0.900   0.099
B M  4YH7A                802    0.148  29.635   0.376   0.004   0.514   0.481
E D  4YH7A                803    0.583  83.981  -0.708   0.018   0.088   0.893
E H  4YH7A                804    0.534  97.135  -0.771   0.005   0.045   0.951
E A  4YH7A                805    0.362  39.859  -2.294   0.004   0.138   0.858
E E  4YH7A                806    0.615 107.493  -1.116   0.004   0.085   0.910
B S  4YH7A                807    0.253  29.698  -1.965   0.004   0.085   0.910
E K  4YH7A                808    0.598 123.091  -0.553   0.019   0.141   0.840
E M  4YH7A                809    0.485  96.948  -1.372   0.021   0.279   0.699
E Y  4YH7A                810    0.343  73.406  -1.537   0.020   0.205   0.775
E A  4YH7A                811    0.463  51.001  -1.605   0.020   0.205   0.775
E T  4YH7A                812    0.477  66.132  -1.589   0.019   0.141   0.840
E S  4YH7A                813    0.381  44.618  -0.673   0.019   0.141   0.840
E P  4YH7A                814    0.533  75.633  -0.998   0.019   0.141   0.840
E Y  4YH7A                815    0.307  65.542  -0.411   0.019   0.141   0.840
E S  4YH7A                816    0.343  40.211  -1.027   0.019   0.141   0.840
E D  4YH7A                817    0.460  66.358  -0.232   0.018   0.088   0.893
E P  4YH7A                818    0.558  79.138  -1.020   0.019   0.141   0.840
E V  4YH7A                819    0.355  54.625  -1.065   0.019   0.141   0.840
E V  4YH7A                820    0.402  61.757  -0.355   0.019   0.141   0.840
B S  4YH7A                821    0.199  23.311  -1.387   0.018   0.047   0.935
E M  4YH7A                822    0.617 123.542  -0.897   0.018   0.019   0.964
E D  4YH7A                823    0.758 109.185  -0.208   0.018   0.019   0.964
E L  4YH7A                824    0.269  49.291  -1.077   0.018   0.019   0.964
E D  4YH7A                825    0.712 102.642   0.225   0.005   0.015   0.979
E P  4YH7A                826    0.367  52.006  -1.074   0.005   0.045   0.951
E Q  4YH7A                827    0.483  86.192   0.250   0.004   0.085   0.910
E P  4YH7A                828    0.463  65.742  -0.793   0.004   0.138   0.858
B I  4YH7A                829    0.143  26.492   0.221   0.004   0.138   0.858
E T  4YH7A                830    0.340  47.103  -0.359   0.004   0.138   0.858
E D  4YH7A                831    0.595  85.812  -0.206   0.004   0.085   0.910
E E  4YH7A                832    0.598 104.540  -1.127   0.005   0.015   0.979
E E  4YH7A                833    0.559  97.622  -1.147   0.018   0.019   0.964
E E  4YH7A                834    0.586 102.304  -0.157   0.018   0.019   0.964
E K  4YH7A                835    0.561 115.336   0.258   0.018   0.019   0.964
B H  4YH7A                836    0.251  45.602  -0.716   0.018   0.019   0.964
B H  4YH7A                837    0.281  51.059  -0.436   0.053   0.043   0.903
E H  4YH7A                838    0.360  65.448  -0.620   0.052   0.084   0.864
E H  4YH7A                839    0.401  73.015  -0.693   0.052   0.084   0.864
E H  4YH7A                840    0.532  96.844  -1.204   0.053   0.043   0.903
E H  4YH7A                841    0.798 145.229  -0.520   0.003   0.003   0.994

Explain the output.  Go back.
```

---
